# Global multiple protein-protein interaction network alignment by combining pairwise network alignments

**DOI:** 10.1186/1471-2105-16-S13-S11

**Published:** 2015-09-25

**Authors:** Jakob Dohrmann, Juris Puchin, Rahul Singh

**Affiliations:** 1 Department of Computer Science, San Francisco State University, San Francisco, CA, USA; 2 Center for Discovery and Innovation in Parasitic Diseases, University of California, San Francisco, San Francisco, CA, USA

**Keywords:** Multiple network alignment, protein-protein interaction network, pair-wise network alignment

## Abstract

**Background:**

A wealth of protein interaction data has become available in recent years, creating an urgent need for powerful analysis techniques. In this context, the problem of finding biologically meaningful correspondences between different protein-protein interaction networks (PPIN) is of particular interest. The PPIN of a species can be compared with that of other species through the process of PPIN alignment. Such an alignment can provide insight into basic problems like species evolution and network component function determination, as well as translational problems such as target identification and elucidation of mechanisms of disease spread. Furthermore, multiple PPINs can be aligned simultaneously, expanding the analytical implications of the result. While there are several pairwise network alignment algorithms, few methods are capable of multiple network alignment.

**Results:**

We propose SMAL, a MNA algorithm based on the philosophy of scaffold-based alignment. SMAL is capable of converting results from any global pairwise alignment algorithms into a MNA in linear time. Using this method, we have built multiple network alignments based on combining pairwise alignments from a number of publicly available (pairwise) network aligners. We tested SMAL using PPINs of eight species derived from the IntAct repository and employed a number of measures to evaluate performance. Additionally, as part of our experimental investigations, we compared the effectiveness of SMAL while aligning up to eight input PPINs, and examined the effect of scaffold network choice on the alignments.

**Conclusions:**

A key advantage of SMAL lies in its ability to create MNAs through the use of pairwise network aligners for which native MNA implementations do not exist. Experiments indicate that the performance of SMAL was comparable to that of the native MNA implementation of established methods such as IsoRankN and SMETANA. However, in terms of computational time, SMAL was significantly faster. SMAL was also able to retain many important characteristics of the native pairwise alignments, such as the number of aligned nodes and edges, as well as the functional and homologene similarity of aligned nodes. The speed, flexibility and the ability to retain prior correspondences as new networks are aligned, makes SMAL a compelling choice for alignment of multiple large networks.

## Introduction

With the advent of high-throughput experimental techniques such as yeast two-hybrid screening [1-3] and co-immunoprecipitation coupled mass spectrometry [4,5] there has been a substantial increase in the data available on protein-protein interactions (PPIs). The experimental data is supplemented by computationally predicted PPIs [6-9]. Put together, a vast amount of PPI data is now accessible through multiple databases [10-13]. Comparative network analysis of PPINs complements traditional sequence and structure based-methods, providing insights into species evolution [14], conserved functional components [15,16], protein function prediction [17,18]. In addition to their role in elucidating a mechanistic understanding of the fundamental biological processes from the molecular to the evolutionary scales [19], PPI-data can also be invaluable in translational contexts, for instance, by explaining mechanisms of infection spread [13,20-23] and through discovery of novel targets, such as dependency factors [24].

The complexity of protein-protein interactions coupled with the volume and noisy nature of PPI data, underline the acute need for automated analysis of PPIs. For computational analysis, the standard way of representing PPI data is through a protein-protein interaction network (PPIN), which is a (possibly disconnected) graph *G *= (*V, E*), where each node represents a protein and each edge denotes an experimentally or computationally determined interaction between the corresponding two proteins. Depending on the detection/prediction method, the edge weights may be binary or real-valued. An important problem in PPIN analysis, much like with traditional sequence-based genomics, is the establishment of correspondences between proteins and interactions across different species. This can be accomplished through PPI network alignment, where, by incorporating network topology, notions of protein similarity and other related data, members of one PPIN are matched with their closest analogues in another PPIN.

In the following, for simplicity, we introduce the basic notions and notations related to network alignment using the pairwise network alignment formulation; the extension of these concepts to the multiple network alignment setting is facile. Formally, given two PPI networks, *G_1 _*= (*V_1_, E_1_*) and *G_2 _*= (*V_2_, E_2_*), where, $\vartheta_{1}\subseteq V_{1}$ and $\vartheta_{2}\subseteq V_{2}$, solving the alignment problem requires finding a correspondence $C:\vartheta_{1}\to \vartheta_{2}$. Intuitively, the objective of any such mapping is to establish correspondences between similar proteins (nodes) and similar intermolecular interactions across the networks. The problem of PPIN alignment was initially tackled as a local alignment problem (that is, the setting considered was with $\vartheta_{1}\subset V_{1}$ and $\vartheta_{2}\subset V_{2}$), where sub-networks with similar topology and/or sequence similarity were identified within the networks being aligned. Later methods have tried to solve the global alignment problem, that is, aligning two PPINs in their entirety ($\vartheta_{1}=V_{1}$ and $\vartheta_{2}=V_{2}$). Both the local and the global alignment problems are known to be *NP*-hard [25,26], and remain active areas of research. Another perspective takes into account the number of networks that need to be aligned, leading to two problem settings: pair-wise network alignment (PNA), involving alignment of two networks at a time and multiple network alignment (MNA), where three or more PPINs have to be aligned to each other. In Additional File 1 (Overview of PPIN alignment algorithms), we classify and summarize the existing methods based on the Cartesian product of the aforementioned formulations and tabulate the results. As can be seen from this table, at the state of the art, the number of pairwise aligners significantly exceeds the number of multiple network alignment algorithms. Furthermore, there are few global multiple network aligners and those that are available tend to rapidly degrade in performance as the number of networks being aligned increases.

The research presented in this papers seeks to address the aforementioned lacunae through the design of a global multiple network aligner called SMAL (**S**caffold-Based **M**ultiple Network **Al**igner, pronounced *small*), which is based on the notion of combining pairwise alignments using a star-like alignment topology with a central "scaffold" PPIN. SMAL allows the use of pairwise network aligners without native MNA implementations (like Pinalog [27] and NETAL [28] for instance), to create MNAs. The star-alignment heuristic, used in SMAL, as is well known, has been applied to other *NP*-hard problems in bioinformatics including multiple sequence alignment and more recently for aligning RNA-seq data [29]. The key features and contributions of SMAL include:

• *Generality*: the star-alignment-like methodology proposed by us can be employed to convert results from any number of global pairwise alignments into a single multiple network alignment. Furthermore, the proposed approach does not restrict the specific pairwise aligner that a biologist may seek to employ.

• *Alignment Persistence*: as networks are added to an already obtained MNA, previously identified alignments are retained.

• *Measure consistency*: For pairwise alignments, a number of statistics have been proposed to quantify the alignment quality. As a corollary to alignment persistence, in the MNAs obtained with the proposed method, the statistics characterizing any constituent pairwise alignment do not change in the multiple alignment.

• *Invariance to alignment order*: It is desirable that a MNA be invariant to the order in which the individual networks are considered. The proposed approach guarantees this property.

• *Conceptual simplicity*: The multiple network alignments obtained with the proposed method can be related to pairwise alignment in conceptually straightforward manners, reducing thereby the cognitive load required for data interpretation by a domain specialist.

• *Low complexity*: The proposed approach has linear-time complexity with respect to the number of networks being aligned. Consequently, as the number of networks that need to be aligned increases, the proposed approach, when compared to competitive methods, yields considerable advantages in terms of time required to obtain a MNA.

• *Alignment quality*: SMAL allows creation of MNAs based on any existing pairwise alignment algorithm. In many cases, this leads to MNAs yielding better results on a given set of measures compared to alignments created by existing native MNA algorithms.

As part of the investigations presented in this paper, we demonstrate the multiple network alignments obtained with the proposed approach by utilizing prior (pairwise) alignments from SMETANA [30], IsoRankN [31], PINALOG [27] and NETAL [28] as inputs. The four methods selected by us are well known or recent and have publicly available implementations. We compare the MNA obtained using our method with those produced by the native multiple network alignment implementations present as part of some of these algorithms.

## Past Work

The problem of PPIN alignment has received significant recent attention. The first PPIN network aligners were primarily designed to identify closely matching subnetworks, rather than solve the global PPIN alignment problem. In and of itself, this is a very challenging problem, as matching two graphs by determining the largest common subgraph is known to be *NP*-hard [25]. Early algorithms, such as PathBLAST [16] and NetworkBLAST [32], used BLAST based search methodology. PathBLAST searched for high-scoring pathway alignments involving linear chains of linked proteins. Proteins in a linear chain from the first input network were paired with their putative homologs in a linear chain in the second input network. Similarity was determined by sequence similarity as determined by BLAST. NetworkBLAST further expanded on this approach by including dense clusters of protein in the search for matching subgraphs. These were followed by MaWISH [33], which adopted an evolutionary model that extended the concepts of match, mismatch, and gap in sequence alignment to that of match, mismatch, and duplication in network alignment, and evaluated similarity between graph structures through a scoring function that accounted for evolutionary events. By contrast, in [34] a statistical model was used to compare the link pattern of each node in the PPIN. Nodes were aligned only if both the sequence and the link pattern were sufficiently similar. The match and split algorithm in [35], is notable for being one of the first to have provable criteria for correctness and efficiency in the context of network alignment. The method Phunkee [36] used the surrounding context of each subgraph within the adjacent network in conjunction with subgraph topology and BLAST data to obtain alignments. Finally, one of the most recent entries into the field is AlignNemo [37], which combined data from PPIN topology and protein homology to iteratively grow local alignments from a seed.

While a local network alignment algorithm seeks to find a set of homologous regions within the two PPINs, a global network alignment seeks to find the best overall alignment between them. That is, a global network alignment algorithm must define a single mapping across all parts of the input. These two problems are, in some sense, analogous to global and local sequence alignment; much like local sequence alignment is used to find conserved functional motifs, local network alignment can be used to find conserved functional components in PPINs (such as pathways, protein complexes etc.) Global sequence alignments, on the other hand, are used to compare whole genomes to understand variations between species; similarly, global PPIN alignment algorithms can be used to compare interactomes across species. However, the global network alignment problem has been shown to be *NP*-hard [26].

While, some of the above local network alignment methods can and have been expanded to produce global alignment, one of the earliest methods to address the global network alignment problem was the eigendecomposition-based method IsoRank [18]. IsoRank conducts its analysis in two steps: it first constructs an eigenvalue problem using PPIN and protein sequence data and solves it to produce a vector *R*, which contains the similarity scores for all protein pairs between the two input networks. In the second step, IsoRank extracts from *R *high-scoring, pairwise, mutually consistent matches and constructs the alignment. Other notable global network alignment algorithms include Graemlin 2.0 [38], which is a hill-climb algorithm that can be trained on a data set to optimize its scoring function, and a relatively large number of algorithms utilizing greedy heuristics, such as PISwap [39], GRAAL [40], MI-GRAAL [14] and variants [41,42]. This problem has also been formulated as a relaxation of a cost function by PATH and GA [43]. In both of these algorithms, the global network alignment problem is expressed as a balance between matching similar protein pairs and having many conserved interactions. The resulting cost function is optimized through two relaxations, one concave and one convex, over doubly stochastic matrices by PATH; and through permutation in the direction of the gradient starting from an initial solution by GA. Finally, one of the most recent efforts, SPINAL [26], is a polynomial time heuristic algorithm that constructs a global alignment in two stages. First, SPINAL constructs pairwise similarity scores though local pairwise neighborhood matching. It then iteratively grows a locally improved solution set to produce the final one-to-one mapping. In both stages SPINAL takes advantage of neighborhood bipartite graphs and the contributors as a common primitive.

More complex than the formulations described above, is the problem of multiple network alignment (MNA), where more than two PPIN network have to be aligned. The computational complexity of MNA grows exponentially as the number of networks increases. MNA algorithms remain relatively rare. Of the few that exist, prominent ones include IsoRankN [31], which is based on spectral clustering on the induced graph of pairwise alignment scores, Submap [44], which utilizes subnetwork mapping followed by vertex selection strategy to extract the mappings from a maximum weight independent set (MWIS), and SMETANA [30], which uses a combination of probabilistic similarity measures to score the nodes and a greedy approach to construct the final alignment.

## Data

In the experiments presented in this paper, we use PPINs from eight different species. These are listed in the following along with the abbreviations we use to refer to them: *Arabidopsis thaliana *(Arabi), *Caenorhabditis elegans *(Celeg), *Drosophila melanogaster *(Droso), *Escherichia coli *(Ecoli), *Homo sapiens *(Human), *Mus musculus *(Mouse), *Rattus norvegicus *(Rat), and *Saccharomyces cerevisiae *(Yeast). The PPINs and corresponding BLAST bit scores are identical to those reported in PINALOG [29], compiled from IntAct [45]. We note that BLAST bit scores were used only for pairs of proteins with a BLAST *E*-value < 10^-5^.

## Methods

The proposed approach begins by determining which of the participating networks can be used as an alignment scaffold (denoted hereafter simply as scaffold or center PPIN) - the network relative to which the entire multiple network alignment is subsequently constructed. The remaining networks are aligned in a pairwise manner with the scaffold PPIN using a pairwise alignment algorithm of choice. In the final step, the pairwise alignments are related to each other. Conceptually, the proposed method is related to the general methodology of star-based methods employed in multiple sequence alignment.

### Definitions and notations

Let *G_1 _*... *G_n _*denote *n *protein-protein interaction networks, where *G*_*i *_= (*V*_*i*_, *E*_*i*_). A global multiple network alignment of *n *graphs can be expressed as a mapping, *Ψ*: *G^n ^*→ *G*, that projects the original graphs onto a structure called the *alignment graph A′ *= (*V′, E′*), such that a cost function for the mapping is optimized. The vertices in the alignment graph represent sets of aligned proteins and its edges correspond to conserved interactions. In the following, variables superscripted with a prime will refer to alignment graphs and unprimed variables will represent elements (graphs, edges and vertices) of specific PPINs. Given a vertex *v′ *∈ *V′ *in the alignment graph, the *vertex alignment cluster *of *v′*, denoted *C*(*v′*) is the set of all nodes mapped to it. Formally, for an alignment involving a set of *m *networks $N=\left\{G_{i},G_{j},\ldots G_{m}\right\}$, the notion of a vertex alignment cluster is formally defined as:

(1) $$C\left(v^{\prime }\right)=\left\{v_{j},\ldots ,v_{l}\right\}:v_{j}\ldots v_{l}\to v^{\prime }\wedge v_{j}\in G_{j},\ldots ,v_{l}\in G_{l},G_{j}\ldots G_{l}\in N$$

That is, for a node in the alignment graph, its vertex alignment cluster consists of a set of proteins from the networks being mapped to it. It follows that, all nodes mapped to a specific node in an alignment graph may be considered to be aligned to each other. Similarly, given a node *v *∈ *V *from any of the original networks, we define the *vertex co-alignment cluster *of *v *as the set of all nodes aligned to node *v *in a multiple network alignment and denote it as B(v). A vertex co-alignment cluster can be accessed using any of its nodes as a key (e.g. {a,b}=B(a)=B(b)). A vertex co-alignment cluster B(v) of a node *v *will at minimum always contain *v *itself. The reader may note that the notion of vertex co-alignment clusters is defined on vertices of PPINs while its dual notion of vertex alignment clusters is defined for vertices of the alignment graph.

The notions of alignment cluster and co-alignment cluster can be extended to edges leading to edge alignment clusters and edge co-alignment clusters (we omit the formal definitions as they are analogous to the ones for vertices). Edges in the alignment graph are induced by the vertex alignment and represent conserved interactions. For a pairwise alignment, for example, a given edge (*u, v*) in a network *G_i _*is said to be conserved in another network *G_j _*if there is an interaction (*s, t*) ∈ *E_j _*such that s∈B(u) and t∈B(v). For the edge (*u, v*) ∈ *E_i _*, its edge co-alignment cluster $E_{ij}\left(u,v\right)$, can be computed as in Eq. (2):

(2) $$E\left(u,v\right)=\left\{\left(s,t\right)B\left(u\right)\times B\left(v\right):\exists G_{j}=\left(V_{j},E_{j}\right):\left(s,t\right)\in E_{j}\right\}$$

In Eq. (2), *j *can denote the index of any of the networks included in the alignment including the network that contains the interaction (*u, v*). In multiple network alignment involving *n *PPINs, generally only very few nodes have correspondences across all *n *species and consequently few edges are conserved across all the *n *species. To model this situation, we use the parameter *k *to consider sets of edges at different levels of conservation. That is, we specifically refer to the set of edges conserved in *k *species when evaluating the alignments.

A given interaction (*u, v*) ∈ *E_i _*is conserved in *k *≤ *n *species, when there are *k-1 *distinct species, such that there exist pairs of nodes (*s, t*) ∈ *E_j _*such that s∈B(u), t∈B(v), with the variable *j *indexing these species.

### Overview of SMAL

The proposed method comprises four major steps: (1) Selection of a network as the scaffold for MNA, (2) Computing pairwise alignments between the scaffold and all other networks, (3) Combining pairwise node alignments with respect to the scaffold, and (4) Computing conserved edges.

#### Selection of the scaffolding network as the center of the star-based MNA

**Figure 1:**
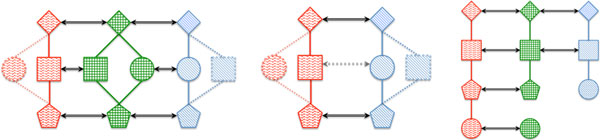
**Network alignment overview**. Similar shapes represent similar proteins that should be aligned. Dotted lines and shapes represent parts that are missing from the respective networks. Arrows represent identified correspondences. The figure on the left shows a network, checkered in the center, which is used to optimally align the other two networks on the left and right. Missing nodes and interactions can be inferred. The figure in the center shows an alignment between only the two outer networks without a central PPIN serving as a scaffold. The alignment will either have to ignore the middle node or accept a suboptimal node alignment. No information about missing nodes and their potential location can be inferred from these two networks alone. Finally, the figure on the right shows an alignment of three networks. The two topmost proteins (diamond and square) are aligned across all three networks and they each interact in their respective PPIN. The interaction ***diamond-square ***is thus conserved in all three species. Since the pentagon has only an aligned protein that also forms an interaction in the two leftmost PPINs, ***square-pentagon ***is conserved in two species only. Pentagon and circle are aligned but since the interaction ***pentagon-circle ***is missing from the middle PPIN, the edge is not considered conserved.

Since selecting an appropriate scaffold has significant influence on the quality of the MNA, the intuition would be to use a network as the center of the star which is most complete, well annotated and evolutionary most similar to the rest (Figure 1). This can be determined based on characteristics such as the maximum number of nodes or edges or the highest count of significant pairwise protein similarities between the networks (e.g. established by BLAST bit scores). By contrast, in certain cases, the specific biological question motivating the MNA, or a researcher's domain knowledge, might dictate which PPIN needs to be chosen as the scaffold.

The proposed algorithm for selecting the scaffold can be described as follows: first, a measure of similarity *S_ij _*defined for a pair of networks is selected. We then pick as the scaffold that specific PPIN for which the sum of *S_ij _*is maximized over all pairs of networks. That is, the network *G_s _*is chosen as the scaffold, if:

(3) $$s=argmax_{i}\left(\underset{j}{\sum }S_{ij}\right)$$

In Eq. (3) *s *is the index of the identified scaffold PPIN. Similarity between a pair of networks can be directly computed, using for example a measure like the Graphlet Degree Distribution agreement [45]. Alternatively, a pairwise alignment can be constructed and a measure of the alignment quality can be used. Such measures derived from pairwise alignments are described in some detail and further investigated in the "Results" section.

#### Pairwise alignments

Given a pairwise network alignment algorithm of choice, the *n-1 *pairwise alignments between the center and the remaining networks *G_sj _*can be computed independently. That is, computation of one alignment has no influence on the results of another alignment. As we will show next, due to this property, the order of alignments in our approach can be arbitrary. Factors that may influence the choice of the alignment algorithm include: characteristics of the obtained alignments such as whether they map proteins in a one-to-one or many-to-many manner, optimization criteria such as maximizing the number of aligned proteins, maximizing conserved interactions or maximizing the size of connected components, computational efficiency, and ease of use. For more details on the characteristics of different pairwise alignment algorithms and implementations, we refer the reader to [47].

#### Combining pairwise node alignments to form the MNA node mappings

From this point onwards, we refer to the co-alignment cluster of a node *v *∈ *V_s _*in the pairwise alignment between networks *G_i _*and *G_j _*as $B_{ij}\left(v\right)$. Let *G_s _*= (*V_s_, E_s_*) denote the scaffold network. Given the terminology introduced above, for each node *v *∈ *V_s_*, B(v) denotes its vertex co-alignment cluster. It is constructed as the union of all co-alignment clusters from the pairwise alignments between the networks $G_{j}\in N$ and the scaffold *G_s_*.

(4) $$B\left(v\right)=\cup B_{sj}\left(v\right)$$

The node alignment obtained with the proposed method can be described as a set of sets containing the alignments for all vertices (proteins) in the scaffold PPIN:

(5) $$V^{*}=\cup \left\{B\left(v\right)\right\}:\forall v\in V_{s}$$

Due to the commutative and associative nature of the union operation over multiple sets, the order in which aligned proteins from the pairwise network alignments are combined can be arbitrary. While the resulting node alignment V^* ^is clearly dependent on the choice of the scaffolding network, the order in which pairwise alignments are themselves computed, or the order in which they are combined, does not matter.

We distinguish two types of pairwise alignments: one-to-one and many-to-many. Methods of the first type aim to find a single correspondence for a given node while methods of the second type can create clusters containing multiple nodes from each of the species that are all related to one another and thus account for phenomena like gene-duplication. The aforementioned distinction, which might inform the choice of the pairwise network alignment algorithm, is preserved in SMAL. If B(v) contains at most one node from PPIN *G_j _*for any node *v *∈ *V_s_*, as would be the case for a one-to-one alignment algorithm, the resulting alignment cluster $B\left(v\right)\in V^{*}$ generated by Eq. (4) will also contain at most one node from each of the aligned species. In this case, each node, including those from the scaffold, will be present in at most one alignment cluster. On the other hand, when multiple nodes of a given species are aligned to a given node *v *∈ *V_s _*in B(v), Eq. (4) ensures that same multiple node alignment is also present in V^*^. Further, if multiple nodes from the scaffold are aligned to one another, this leads to node duplication, vide infra.

The combination of aligned nodes, as described above, induces a relationship, which we term as *weak correspondence transitivity*. As an explanation, consider two networks *G_a _*and *G_b _*being aligned to a scaffold *G_s_*. Further, let node *a *∈ *V_a _*and *b *∈ *V_b _*correspond to the node *u *∈ *V_s _*based on their respective pairwise alignments. Then B(a)={u,a}, B(b)={u,b}, and B(u)={u,a,b}. Such a grouping implies a putative correspondence between nodes *a *and *b*. However, not all of these putative alignments may be found in a multiple network alignment. This is either due to noise in the data or because strict transitivity of the correspondences does not hold. We present results of our studies of this effect in detail in the "Results" section.

#### Computing conserved edges

For each edge (*u, v*) in the scaffold *G_s _*of a MNA, the set of associated conserved edges is given by its edge co-alignment cluster defined by Eq. (2). The following equation can be formulated alternatively as shown in Eq. (5), or implemented directly.

(6) $$E\left(u,v\right)=\left\{\left(k,l\right)\in B\left(u\right)\times B\left(v\right)|\exists t:\;\left(k,l\right)\in E_{t}\right\}$$

That is, the conserved edges relative to a given edge in the scaffold PPIN in the MNA can be directly computed from the node alignment set V^* ^defined in Eq. (5). Analogous to the node alignment, the set of induced edges as derived by the proposed method then can be described as:

(7) $$\{E^{*}=\cup \left\{E\left(u,v\right)\right\}:\forall \left(u,v\right)\in E_{s}$$

As with the node alignment, the conserved edges will depend on the choice of the center PPIN but will otherwise be independent from the order in which networks are aligned pairwise or combined in our star-based approach.

#### Differences to established MNA algorithms

In network alignments in general, a given vertex from any of the original networks is either dropped (not aligned to any other node) or included in the alignment graph *V' *exactly once.

Since SMAL maps alignment clusters from pairwise alignments onto a central PPIN, proteins can be duplicated. To elucidate, let's assume a scenario where the scaffold PPIN *G_s _*is aligned relative to two networks *G_a _*and *G_b_*. Consider the following two alignment clusters from pairwise alignments for given nodes *u, v, w *∈ *V_s_, a *∈ *V_a _*and *b *∈ *V_b_*:

$$\begin{array}{c} B_{sa}\left(u\right)=\left\{u,v,a\right\}=B_{sa}\left(v\right)\\ B_{sb}\left(u\right)=\left\{u,w,b\right\}=B_{sb}\left(w\right) \end{array}$$

This will result in the following three alignment clusters in a star-based MNA as proposed here:

$$\begin{array}{c} B\left(u\right)=B_{sa}\left(u\right)\cup B_{sb}\left(u\right)=\left\{u,v,w,a,b\right\}\\ B\left(v\right)=\left\{v,u,a\right\}\\ B\left(w\right)=\left\{w,u,b\right\} \end{array}$$

On the other hand, since the alignment graph of SMAL V^* ^contains only alignment clusters for the nodes of the center PPIN, some correspondences established by native multiple network alignments are not considered. Let there be nodes *a *∈ *V_a_, b *∈ *V_b _*that correspond when aligning *G_s_, G_a _*and *G_b _*with a native multiple network alignment algorithm but neither corresponds to any vertex in the scaffolding PPIN. That is, there exists an alignment cluster B(a)={a,b,X}=B(b), where *X *is a set of nodes that are not part of the center PPIN or the empty set. Such correspondences would not be included by SMAL. Expanding SMAL to such correspondences could be achieved by considering all pairwise alignments (as opposed to only alignments between a center PPIN and the remaining networks) and merging resulting alignment clusters with V^*^.

#### Implementation and complexity

Pseudo-code 1: Method outline

1 Designate scaffold PPIN *G_s_*

# Obtain pairwise alignments with the scaffold PPIN using a method of choice.

2 For all remaining networks *G_j_*:

3     *G_sj _*← pairwise_alignment(*G_s_, G_j_*)

# Create node alignment

4 Initialize V^* ^= Ø

5 For each node of *G_s_, v *∈ *V_s_*:

6     Initialize $B\left(v\right)=\left\{v\right\}$

7     For each pairwise alignment *G_s_, G_j_*:

8         $B\left(v\right)\leftarrow B\left(v\right)\cup B_{sj}\left(v\right)$

9     $V^{*}\leftarrow V^{*}\cdot B\left(v\right)$ # concatenate sets

# Compute induced edges

10 Initialize E^* ^= Ø

11 For each edge of *G_s_*, (*u, v*) ∈ *E_s_*:

12     Initialize $E\left(u,v\right)=\left\{\left(u,v\right)\right\}$

13     For each pair $\left(k,l\right)\in B\left(u\right)\times B\left(v\right):$

14         if (*k, l*) form an edge, e.g. $*t *: (*k, l*) ∈ *E_t_*:

15             $E\left(u,v\right)\leftarrow E\left(u,v\right)\cup \left(k,\;l\right)$

16     $E^{*}\leftarrow E^{*}\cdot E\left(u,v\right)$ # concatenate sets

In the pseudo-code, selection of the scaffold is summarized in line 1. Different approaches of varying complexities have been mentioned and will be evaluated in the "Results" section. In terms of computational complexity, scaffold selection based on domain expertise does not incur a computational cost. A simple heuristic like the number of associated BLAST bit scores above a certain *E*-Value for a given PPIN is also extremely fast ($O\left(n\right)$, where *n *is the number of networks). Selection based on a similarity measure between all pairs of networks has complexity $O\left(n^{2}\times O\left(\phi \right)\right)$, where $O\left(\phi \right)$ is the complexity of the applied similarity measure. The approach using measures over pairwise alignments outlined in the Methods section can be further broken down to $O\left(n^{2}\times \left(O\left(\varphi \right)+O\left(\mu \right)\right)\right)$, where $O\left(\varphi \right)$ is the complexity of the pairwise alignment algorithm and $O\left(\mu \right)$ the complexity of the measure over the alignment. For our node-based measures, $O\left(\mu \right)=O\left(\varrho \left|V_{s}\right|\right)$, where ϱ=max(|B(v)|); *v *∈ *V_s_*, the maximum number of nodes in an alignment cluster in V^*^. The actual size of  ϱ depends on the alignment algorithm. For one-to-one alignment algorithms, we know that ϱ≤n. For many-to-many algorithms, no non-trivial boundary can be established.

Once a scaffolding PPIN is selected, (n-1) pairwise alignments are computed (lines 2 and 3). This step has complexity $O\left(n\times O\left(\varphi \right)\right)$ though no computation might be necessary if pairwise alignments have already been created during the scaffold-selection process.

Creation of the node alignments (lines 4 to 9) has complexity $O\left(n\left|V_{s}\right|\right)$. The alignment clusters B(v) are sets of distinct nodes that get extended in each iteration of line 8. V^* ^consists of a list of such sets of elemental nodes. The structure is implemented as a dictionary of sets where each key is a node *v *∈ *V_s _*and the corresponding value represents B(v).

The last step (lines 10 to 16) is not specific to our approach and most of the established alignment algorithms just omit it. It can be applied to any kind of node alignment. We include it in our algorithm since providing insights into conserved interactions is essential for many of the research questions that motivate MNAs in the first place. It also provides insights into the quality of the alignment via various measures as described later. Complexity of this last step has an upper bound of $O\left(\varrho^{2}\left|E_{s}\right|\right)$.

Overall, the complexity of SMAL with selection of the center PPIN via measures over pairwise alignments is $O\left(n^{2}\times \left(O\left(\varphi \right)+O\left(\mu \right)\right)+n\left|V_{s}\right|+\varrho^{2}\left|E_{s}\right|\right)$. When the center is selected manually based on domain knowledge or any other accessible proxy as outlined above, complexity is reduced to $O\left(n\times O\left(\varphi \right)+n\left|V_{s}\right|+\varrho^{2}\left|E_{s}\right|\right)$. By far the most expensive step is computation of the pairwise alignments, that is $O\left(\varphi \right)\gg O\left(\left|V_{s}\right|\right)$ and $O\left(\varphi \right)\gg O\left(\left|E_{s}\right|\right)$.

#### Comparison between SMAL and native MNAs

To compare a native MNA generated by a given MNA algorithm to a SMAL MNA, where the pairwise alignments have been generated by the same algorithm, we first have to relate the native MNA to our chosen scaffold PPIN. This can be achieved by only retaining those node clusters that contain a protein from the designated scaffold PPIN and by duplicating clusters containing more then one scaffolding node (see pseudo-code 2 in Additional file 2).

### Measures for assessment

**Figure 2:**
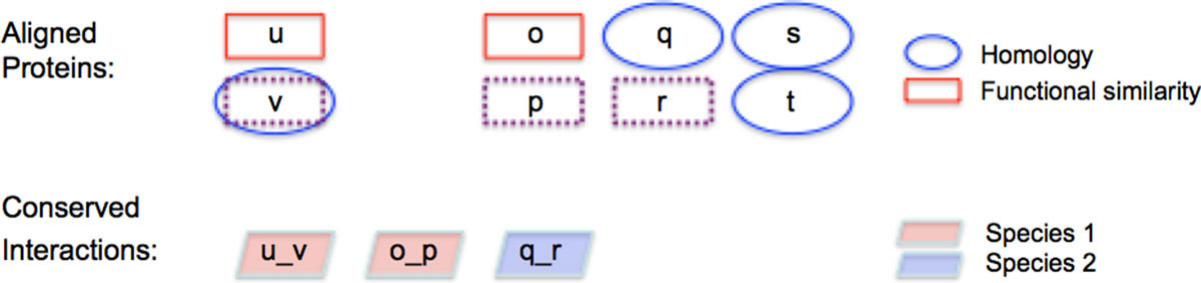
**Examples and explanations of measures**. This figure shows two node alignment clusters, B(u)={u,o,q,s} and B(v)={v,p,r,t}, and a corresponding cluster of induced edges E(u,v)={(o,p),(q,r)}. Note that not all aligned nodes induce edges in general; ***s ***and ***t ***in this example are assumed to not interact in any of the aligned PPINs. The pairs ***u***:***o***, ***v***:***p***, ***v***:***r ***and ***p***:***r ***all contribute to the number of aligned nodes with high functional similarity ***NF ***but only ***u***:***o***, ***v***:***p ***and ***v***:***r ***count towards the scaffold centric measure ***NF_s_***. ***p***:***r ***does not contribute to ***NF_s _***as the pair does not contain ***V***, the root node of the alignment cluster. Equally, ***q***:***s ***and ***v***:***t ***contribute to the number of aligned homologous nodes ***NH ***while only ***v***:***t ***adds to ***NH_s_***, the scaffold centric version of the same measure. Each of the node alignment clusters contributes a count of 3 to ***NA_s_***, the number of nodes that are aligned to the scaffold nodes, and 6 to the number of all possible pairs of aligned nodes ***NA***. Considering the conserved interaction (***o***, ***p***), since both endpoints are functionally similar to the endpoints of the interaction between the scaffolding nodes spanning the respective alignment clusters (e.g. ***u***, ***o ***and ***v***, ***p ***are functionally similar), it contributes to ***EF_s _***(and also to ***EF***). Note that ***u***, ***v ***(and thus ***o***, ***p***) do not need to be functionally similar to each other. The fact that ***s ***and ***t ***are homologous does not lead to any contributions. The cluster of induced edges adds 2 to the total number of interactions aligned to the scaffold interactions ***EA_s _***and 3 to the same measure accounting for all possible pairs of aligned interactions ***EA***. Even though there are three conserved interactions, this cluster contributes to ***EA-2 ***only since the three edges belong to only two distinct species.

Since there is no single gold standard for evaluating biological network alignments, we use a number of different measures in our analysis. In addition to evaluating the overall quality of the alignments, we investigate the extent to which correspondences implied by combining pairwise alignments are valid biologically. For this purpose, we define two types of measures: Measures designated with the subscript *s*, which only evaluate correspondences with the scaffold. In other words, for each node *v *∈ *V_s _*only the pairs *v, u *: u∈B(v) and for each edge *e *∈ *E_s _*only the pairs *e, f *: f∈E(e) are taken into account in these measures. Thus, these measures represent a baseline as to how the pairwise alignments perform on the given data. The measures without subscript on the other hand evaluate all correspondences, that is for all $B\left(v\right)\in V^{*}$, consider all pairs *s*, t∈B(v) and for all $E\left(e\right)\in E^{*}$, all *f*, g∈E(e) respectively. These measures also capture putative alignments (Figure 2). Since alignment clusters containing more than one node or edge from the scaffold PPIN are associated with each contained scaffolding node or edge, such clusters are counted multiple times. We investigated this effect and computed measures for distinct clusters (without double counting). We determined that the key findings of this investigation are the same for both approaches.

#### Aligned nodes with high functional similarity (*NF*) or homology (*NH*)

To measure how well the biological functionality of the proteins is reflected in the alignment graph, we define an auxiliary function.

(8) $$F\left(k,l\right)=\left\{\begin{array}{c} 1,if\;nodes\;k\;and\;l\;have\;functional\;similarity\;score\;>0.5\\ 0,else \end{array}\right)$$

**Figure 3:**
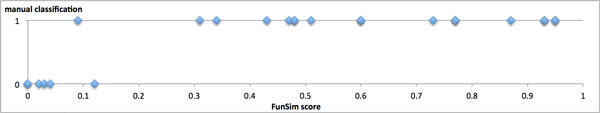
**funSim scores versus manual classification in RNA Polymerase complex**. The y-axis represents manual classification (1 signifying a biologically relevant match). A threshold of around 0.2 appears adequate for capturing biologically relevant protein correspondences in this case.

Functional similarity scores for each pair of aligned proteins are according to the funSim score in FunSimMat [48]. The funSim score combines similarity scores with respect to both involvement in biological processes and molecular function for a pair of proteins. Scores reach from 0 (no similarity) to 1 (maximum similarity) and are computed based on semantic similarity of the GO terms of the two proteins and their respective probabilities. Manual review appears to suggest that this threshold could be lowered further to capture more relevant protein-protein correspondences without significantly increasing the number of false positives (Table 1 Figure 3). The threshold of 0.5 has been used in the literature to evaluate alignments [27] and is thus used here for easier comparison.

**Table 1:** funSim scores and homolgene ID matches in RNA Polymerase complex compared to manual classification

Scaffold	Gene	Aligned	Gene	FunSim	Homologene	Manual
P52435	Polr2j	A1Z9J6	mRpL53	0	mismatch	mismatch
P19387	POLR2C	Q9V3G9	BACR37P7.5	0	mismatch	mismatch
P53803	Polr2k	Q9VG44	CG6225	0.02	mismatch	mismatch
P52434	POLR2H	Q9VKS9	CG18284	0.03	mismatch	mismatch
P62487	POLR2G	Q9VJB3	CG5681	0.04	mismatch	mismatch
P62875	POLR2l	P14284	REV3	0.09	mismatch	match
P19388	POLR2e	Q8SXU3	CG8207	0.12	mismatch	mismatch
O15514	POLR2d	P20433	RPB4	0.31	match	match
P52434	POLR2H	P20436	RPB8	0.34	match	match
P19387	POLR2C	P16370	RPB3	0.43	match	match
P62487	POLR2G	P34087	RPB7	0.47	match	match
P52435	Polr2j	P38902	RPB11	0.48	match	match
P19388	POLR2e	P20434	RPB5	0.48	match	match
P24928	POLR2a	P04050	RPO21	0.51	match	match
P61218	POLR2F	P20435	RPO26	0.6	mismatch	match
P30876	POLR2B	P08518	RPB2	0.6	match	match
O15514	POLR2d	Q9VEA5	Rpb4	0.73	match	match
P24928	POLR2a	P04052	RpII215	0.77	match	match
P61218	POLR2F	Q24320	RpII18	0.77	match	match
P30876	POLR2B	P08266	RpII140	0.87	match	match
P19387	POLR2C	P97760	Polr2c	0.93	match	match
P62875	POLR2l	Q9VC49	Rpb10	0.93	match	match
P30876	POLR2B	Q8CFI7	Polr2b	0.95	match	match

A threshold of 0.2 for *NF *would better cover the biologically relevant protein mappings than the currently used value of 0.5. While *NH *has both high coverage and accuracy in this complex, it is in general also missing many valid correspondences. *NForH *is most consistent with manual classification.

To capture how well the alignment recovers the evolutionary relationship of the nodes in the input networks, we define a set of related measures accounting for pairwise homologous proteins based on another auxiliary function.

(9) $$H\left(k,l\right)=\left\{\begin{array}{c} 1,\;if\;k,l\;share\;a\;homologene\;group\;ID\\ 0,else \end{array}\right)$$

In Eq. (9), the homologene group identifiers for each protein are retrieved from the NCBI homologene repository. Often when the *NH *and *NF *measures disagree, the reason is either incomplete (missing) data or, in the case of *NF*, the specification of a threshold value that is overly restrictive for identifying biologically relevant mappings. We introduce a combined measure that counts all nodes that are either functionally similar or homologous. As outlined above, we define two variations of each measure.

(10) $$NF_{s}=\sum_{u\in V_{s}}\sum_{t\in B\left(u\right),t\neq u}F\left(u,t\right)$$

(11) $$NF=\sum_{u\in V_{s}}\sum_{\left\{s,t\right\}\in B\left(u\right),s\neq t}F\left(s,t\right)$$

(12) $$NH_{s}=\sum_{u\in V_{s}}\sum_{t\in B\left(u\right),t\neq u}H\left(u,t\right)$$

(13) $$NH=\sum_{u\in V_{s}}\sum_{\left\{s,t\right\}\in B\left(u\right),s\neq t}H\left(s,t\right)$$

(14) $$NForH_{s}=\sum_{u\in V_{s}}\sum_{t\in B\left(u\right),t\neq u}min\left(1,F\left(u,t\right)+H\left(u,t\right)\right)$$

(15) $$NForH=\sum_{u\in V_{s}}\sum_{\left\{s,t\right\}\in B\left(u\right),s\neq t}min\left(1,F\left(s,t\right)+H\left(s,t\right)\right)$$

#### Number of aligned nodes (*NA*) and derived measures of precision

The number of aligned nodes is considered only to normalize other measures. Dividing by *NA *allows for establishing a measure of precision since *NA *captures all aligned nodes (e.g. *true positives *and *false positives*) while other metrics like *NF *or *NH *can be considered the *true positives *according to their specific biological perspective. For normalization of the two different varieties for each metric we define

(16) $$NA_{s}=\sum_{u\in V_{s}}\sum_{t\in B\left(u\right),t\neq u}1=-\left|V_{s}\right|+\sum_{u\in V_{s}}|B\left(u\right)|$$

(17) $$NA=\sum_{u\in V_{s}}\sum_{\left\{s,t\right\}\in B\left(u\right),s\neq t}1$$

Eq. (16) specifies the number of nodes aligned to the nodes of the center PPIN. The scaffolding nodes themselves are excluded. This gives *NA_s _*the same range as *NF_s_, NH_s _*and *NForH_s _*and allows for normalization of those measures in the range [0 1].

#### Conserved interactions with functionally similar (*EF*) or homologous (*EH*) endpoint proteins

Conserved interactions in general are a relevant measure of alignment quality (see number of aligned edges, *EA*, below). *EF *is a biologically motivated variation of this measure where the two pairs of interacting proteins, the endpoints of the edges, are considered as well. Only interactions where the aligned endpoint proteins are pairwise functionally similar as defined in Eq. (8) are counted towards this measure.

(18) $$EF_{s}=\sum_{\left(u,v\right)\in E_{s}}\sum_{\left(s,t\right)\in E\left(u,v\right),s\in B\left(u\right),t\in B\left(v\right),\left(s,t\right)\neq \left(u,v\right)}F\left(u,s\right)*F\left(v,t\right)$$

(19) $$EF=\sum_{\left(u,v\right)\in E_{s}}\sum_{\left\{\left(q,r\right),\left(s,t\right)\right\}\in E\left(u,v\right),\left\{q,s\right\}\in B\left(u\right),\left\{r,t\right\}\in B\left(v\right)}F\left(q,s\right)*F\left(r,t\right)$$

Analogous, based on Eq. (9) we define

(20) $$EH_{s}=\sum_{\left(u,v\right)\in E_{s}}\sum_{\left(s,t\right)\in E\left(u,v\right),s\in B\left(u\right),t\in B\left(v\right),\left(s,t\right)\neq \left(u,v\right)}H\left(u,s\right)*H\left(v,t\right)$$

(21) $$EH=\sum_{\left(u,v\right)\in E_{s}}\sum_{\left\{\left(q,r\right),\left(s,t\right)\right\}\in E\left(u,v\right),\left\{q,s\right\}\in B\left(u\right),\left\{r,t\right\}\in B\left(v\right)}H\left(q,s\right)*H\left(r,t\right)$$

With the same reasoning we presented for the combined node-based measure *NForH *we define corresponding interaction-based measures as follows

(22) $$EForH_{s}=\sum_{\left(u,v\right)\in E_{s}}\sum_{\left(s,t\right)\in E\left(u,v\right)}min\left(1,\left(F\left(u,s\right)+H\left(u,s\right)\right)*\left(F\left(v,t\right)*H\left(v,t\right)\right)\right)$$

(23) $$EForH=\sum_{\left(u,v\right)\in E_{s}}\sum_{\left\{\left(q,r\right),\left(s,t\right)\right\}\in E\left(u,v\right)}min\left(1,\left(F\left(q,s\right)+H\left(q,s\right)\right)*\left(F\left(r,t\right)+H\left(r,t\right)\right)\right)$$

where *q*, s∈B(*u*); *r*, t∈B(v), (*s, t*) ≠ (*u, v*) and (*q, r*) ≠ (*s, t*).

#### Number of conserved edges (*EA*)

The number of conserved edges in the alignment graph reflects how well the aligned proteins capture the topology and biological processes expressed in the input networks and allow evaluation of the quality of the alignment independent of biological measures like functional similarity or homology.

(24) $$EA_{s}=\sum_{e\in E_{s}}\sum_{d\in E\left(e\right),d\neq e}1=-\left|E_{s}\right|+\sum_{e\in E_{s}}|E\left(e\right)|$$

(25) $$EA=\sum_{e\in E_{s}}\sum_{\left\{c,d\right\}\in E\left(e\right)}1$$

Analogous to *NA, EA *can also be used to derive biologically inspired precision measures on edges.

#### Number of interactions conserved in at least *k *distinct species (*EA-k*)

In addition to the total number of conserved interactions *EA*, we define the number of interactions that are conserved in at least *k *species *EA-k *as the number of edges (*u, v*) ∈ *E_s _*that have induced edges from at least *k*-*1 *non-scaffold species associated with them. An edge with one induced edge from a different species would count towards *EA-2*. An edge with induced edges from two additional distinct species would contribute to *EA-2 *but also count towards *EA-3 *and so forth. The tautological *EA-1 = *|*E_s_*| does not provide information for characterizing an alignment.

## Results

### Effect of the scaffold selection on the SMAL MNA

To demonstrate measure consistency, we compared the performance of SMAL to that of pairwise network aligners. To estimate pairwise performance, for each algorithm, we computed all pairwise alignments and took the sum of each measure across all alignments involving a given algorithm and species. The highest, and second highest scoring species for each algorithm and measure is presented in Table 2. To generate a comparable table for SMAL, we produced a SMAL alignment for each algorithm in turn using each of the eight species PPINs as the scaffold, and computed the same measures for each of these MNAs. The scores of the highest and second highest scoring MNAs together with the corresponding scaffold species for each algorithm are presented in Table 3.

**Table 2:** Pairwise algorithm performance

max-scores (sum over pairs)	*NA_s_*	*NF_s_*	*NH_s_*	*NForH_s_*	*EA-2*	*EA_s_*	*EF_s_*	*EH_s_*	*EForH_s_*
**IsoRankN highest**	**Human** 20054	**Human** 8264	**Human** 2874	**Human** 8997	**Human** 1865	**Arabi** 7067	**Arabi** 1678	**Human** 213	**Arabi** 1767
IsoRankN second	*Droso* 17968	*Mouse* 7047	*Mouse* 2411	*Mouse* 7633	*Yeast* 1630	*Yeast* 5586	*Yeast* 1370	*Mouse* 162	*Yeast* 1374

**SMETANA highest**	**Human** 62172	**Human** 32028	**Human** 6774	**Human** 33942	**Human** 7099	**Human** 32344	**Arabi** 17783	**Human** 996	**Arabi** 17881
SMETANA second	*Droso* 57266	*Droso* 27410	*Droso* 5290	*Droso* 29144	*Yeast* 5082	*Arabi* 25013	*Yeast* 7185	*Yeast* 741	*Yeast* 7477

**PINALOG highest**	**Human** 17764	**Human** 7005	**Human** 4385	**Human** 8130	**Yeast** 10179	**Yeast** 10202	**Human** 1267	**Human** 832	**Human** 1516
PINALOG second	*Droso* 17173	*Droso* 5711	*Mouse* 2984	*Droso* 6586	*Human* 8866	*Human* 8870	*Yeast* 1024	*Yeast* 477	*Yeast* 1220

**NETAL highest**	**Human** 27869	**Human** 796	**Droso** 3	**Human** 797	**Human** 37852	**Human** 37852	**Human** 123	**Droso** 1	**Human** 123
NETAL second	*Droso* 27283	*Droso* 448	*Celeg*, *Human* 1	*Droso* 449	*Droso* 23888	*Droso* 23888	*Droso*, *Mouse* 29	-,0	*Droso* 30

For a given algorithm and measure, the species PPIN with the highest (bold) and second highest (underlined) score sum over all pairwise alignments is presented, along with the score obtained. The highest scoring network for a given algorithm and measure of choice is the one our methodology would designate as the scaffolding PPIN. Note that NETAL found only one conserved interaction with endpoint proteins homologous to their aligned counterparts (*EH*). We thus only present one value in the corresponding cell.

**Table 3:** Performance of SMAL

max-scores (SMAL)	*NA*	*NF*	*NH*	*NForH*	*EA-2*	*EA*	*EF*	*EH*	*EForH*
**IsoRankN first**	**Human** 64812	*Mouse* 22464	**Human** 4676	*Mouse* 23598	**Human** 1665	*Yeast* 68329	**Arabi** 27733	Arabi 749	**Arabi** **28031**
IsoRankN second	Celeg 62677	**Human** 21849	*Mouse* 4459	**Human** 23090	*Yeast* 1458	**Arabi** 66757	Human 9038	**Human** 380	Human 9107

**SMETANA first**	**Human** 349645	**Human** 165645	Celeg 19876	**Human** 170367	**Human** 5328	**Human** 1038837	**Arabi** 809249	Arabi 5420	**Arabi** 809594
SMETANA second	*Droso* 313439	*Droso* 139636	**Human** 16432	*Droso* 144107	*Yeast* 3741	*Arabi* 1020591	Human 268958	**Human** 4155	Human 269553

**PINALOG first**	**Human** 35996	**Human** 12093	**Human** 6873	**Human** 13930	**Yeast** 8541	**Yeast** 12174	**Human** 1509	**Human** 971	**Human** 1815
PINALOG second	Yeast 35970	*Droso* 11325	*Mouse* 5217	*Droso* 12749	*Human* 7387	*Human* 10560	*Yeast* 1347	*Yeast* 644	*Yeast* 1595

**NETAL first**	*Droso* 67555	**Human** 1523	**Droso** 7	**Human** 1526	**Human** 21947	**Human** 63412	**Human** 173	**Droso**, Human 1	**Human** 174
NETAL second	**Human** 66700	*Droso* 1437	*Human* 5	*Droso* 1440	*Droso* 14318	*Droso* 39302	*Droso* 56	-, 0	*Droso* 57

For a given algorithm and measure, the scaffold PPIN with the highest and second highest score obtained through SMAL MNA is presented, along with the achieved score. For easy comparison with Table 2, the species with highest and second highest pairwise sum score are bold and underlined, respectively. For every measure and algorithm tested, the highest scoring species from Table 2 is always either the highest or second highest scoring species in Table 3 (this table), demonstrating measure consistency of SMAL and validating the proposed scaffold selection strategy.

We observe that choice of algorithm and scaffold network greatly affect the alignment results. For instance, Human, Yeast and Drosophila networks, which contain a large number of proteins and interactions (Table 4), receive maximum scores when summing up over their pairwise alignments in almost all of the measures (Table 2). Arabidopsis, which is a small but highly clustered network, scores high on edge-based measures for alignment algorithms (IsoRankN and SMETANA), which can compute many-to-many node alignments (Table 2). This is in line with the suggested heuristic of using simple network statistics like the number of nodes and edges as a proxy for selecting the scaffold put forward in the "Methods" section.

**Table 4:** PPIN Overview

Species	Proteins	Interactions	BLAST (inter-species)	Clustering Coefficient
Arabidopsis	2651	5235	73221	**0.133**
C. elegans	4305	7746	135907	0.023
Drosophila	8374	25610	261864	0.023
E. coli	2818	13841	15401	0.097
Human	**9003**	34935	**340626**	0.095
Mouse	2897	4372	171737	0.129
Rat	1150	1305	61318	0.075
Yeast	5674	**49830**	107616	0.127

The BLAST column shows the number of inter-species protein pairs where one protein is part of the species in the given row and for which we have a BLAST bit score recorded in our data set. Bold typeface indicates the largest value in a column.

Comparing Table 2 and Table 3 we observe that a given choice of algorithm and measure will yield a similar species ranking. We term this effect measure consistency, whereby knowledge of an algorithm's pairwise performance on a given dataset can be extrapolated to estimate the expected performance of said algorithm in a SMAL alignment.

### Precision of implied SMAL mappings compared to native MNAs

As mentioned in the "Methods" section, correspondences between nodes mapped to the same vertex in pairwise alignments with the scaffold are implied when creating the SMAL MNA. To evaluate this transitivity assumption, we measure the biological significance of the putative alignments made by SMAL. This is achieved by calculating the following measure of precision:

(26) $$Precision\left(NForH\right)=\left(NForH-NForH_{s}\right)/\left(NA-NA_{s}\right)$$

Eq. (26) represents the ratio of biologically significant implied node alignments and the total number of implied node alignments. The same equation can be applied to other measures, such as *NF, NH, EF, EH *or *EForH *to obtain corresponding measures of precision. We compare and present the relative change in precision between SMAL and native MNAs.

(27) $$\left(Precision{\left(NForH\right)}_{SMAL}-Precision{\left(NForH\right)}_{native}\right)/Precision{\left(NForH\right)}_{native}$$

**Figure 4:**
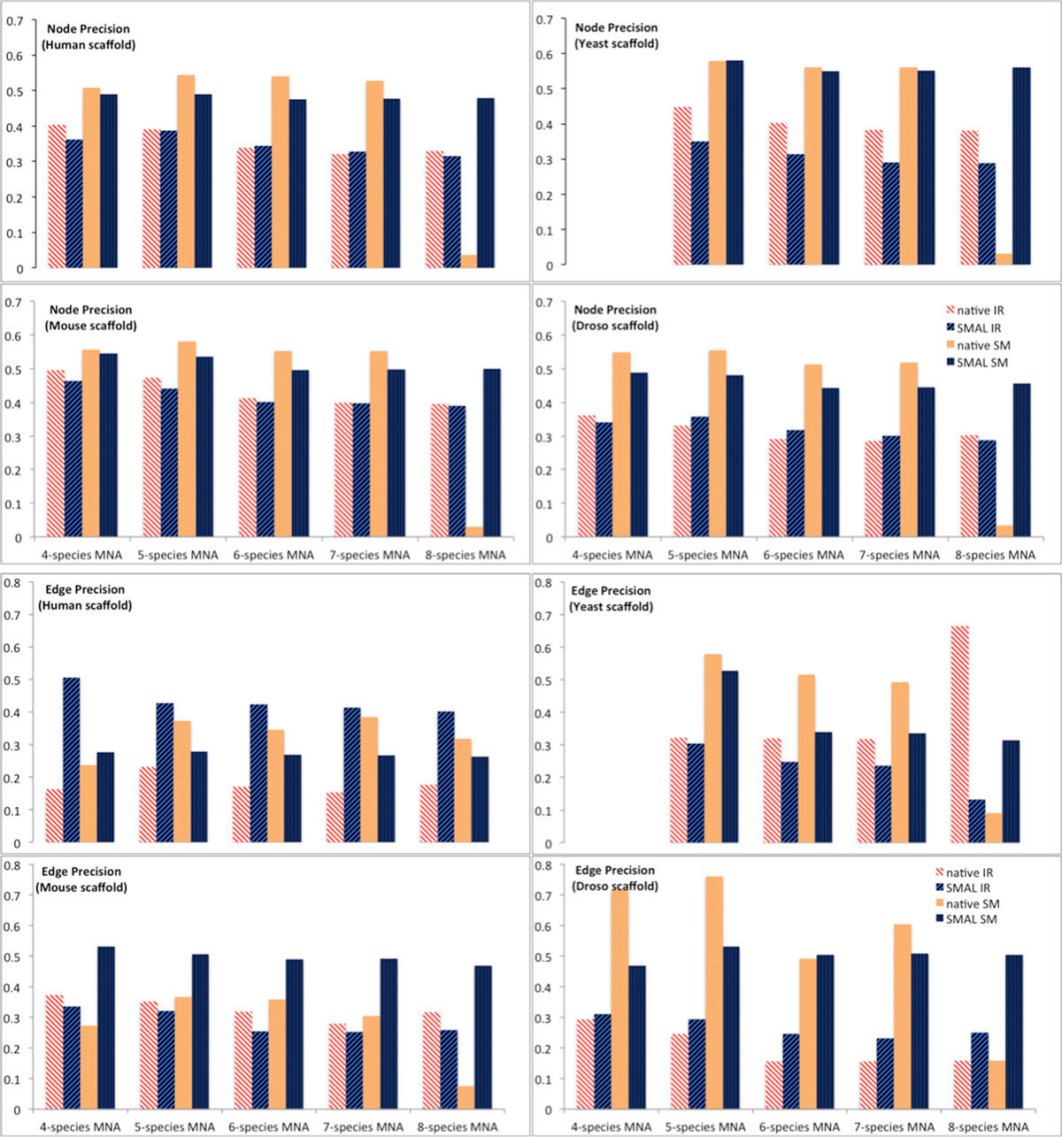
**Relative precision of alignments**. Top two rows show relative precision of node mappings on the y-axis: (NForH - NForHs)/(NA - NAs) in SMAL and native MNAs for different scaffold PPINs. The bottom two rows show relative precision of putative edge alignments (EForH - EForHs)/(EA - EAs). This latter measure is characterized by higher variability when compared to the former. In edge precision, using Human PPIN as the scaffold, SMAL consistently outperforms the native IsoRankN MNA. So does SMAL with SMETANA using mouse as the scaffold. In other cases, the native MNA performs better. PINALOG and NETAL, which do not have native MNA implementations, are not shown in these graphs.

While there is a great deal of variability in the precision of MNA alignments produced by different algorithms as computed by equation (27) (see Figure 4), the precision of SMAL is on average -6.5% of the native MNA implementation for a given algorithm (Table 5) when excluding 4-species MNAs, which are missing Yeast and ignoring 8-species MNAs where SMETANA performs exceptionally poorly. Including all MNAs, SMAL is on average more precise than native MNA implementations with a relative change of precision of 143% on this data set. In the worst case, SMAL can have up to 24.5% worse precision than the native MNA. Thus, for situations when a native MNA implementation performs poorly (e.g. SMETANA with 8-species), or when native MNA implementations do not exist (see "Case studies"), SMAL becomes a particularly useful alternative. Also, for certain measures and scaffolds, SMAL outperforms existing algorithms by significant margins (Figure 4). Finally, we find that the simple transitivity assumption made by SMAL holds up reasonably well (-6.5% loss of precision on this dataset as outlined above) considering the largely reduced complexity compared with the native MNA implementations investigated here.

**Table 5:** Precision in SMAL and native MNA implementations

NForH	IsoRankN	SMETANA	IsoRankN	SMETANA	IsoRankN	SMETANA	IsoRankN	SMETANA
Relative change in precision	Droso	Droso	Human	Human	Mouse	Mouse	Yeast	Yeast
4-species	-5.82%	-11.13%	-10.21%	-3.74%	-6.44%	-2.03%	NA	NA
5-species	8.24%	-13.41%	-1.20%	-9.97%	-6.67%	-7.83%	-21.59%	0.24%
6-species	9.27%	-13.67%	1.89%	-12.02%	-2.74%	-10.39%	-21.83%	-1.79%
7-species	5.07%	-13.99%	2.79%	-9.50%	-0.34%	-9.86%	-24.33%	-1.52%
8-species	-5.05%	1211.83%	-4.18%	1239.56%	-1.17%	1534.16%	-24.44%	1697.95%

Comparison of alignment precision with regards to the *NForH *measure as expressed by Equation (26). Values represent the relative change in precision using the native implementation as the reference according to Equation (27). Positive values in the table correspond to alignments where SMAL was more precise than the native implementation. Negative values appear when SMAL was less precise. Since Yeast was not part of the 4-species MNA, no value is presented in the respective cells.

### Case studies

#### PINALOG: MNAs with a high ratio of aligned homologous proteins

**Figure 5:**
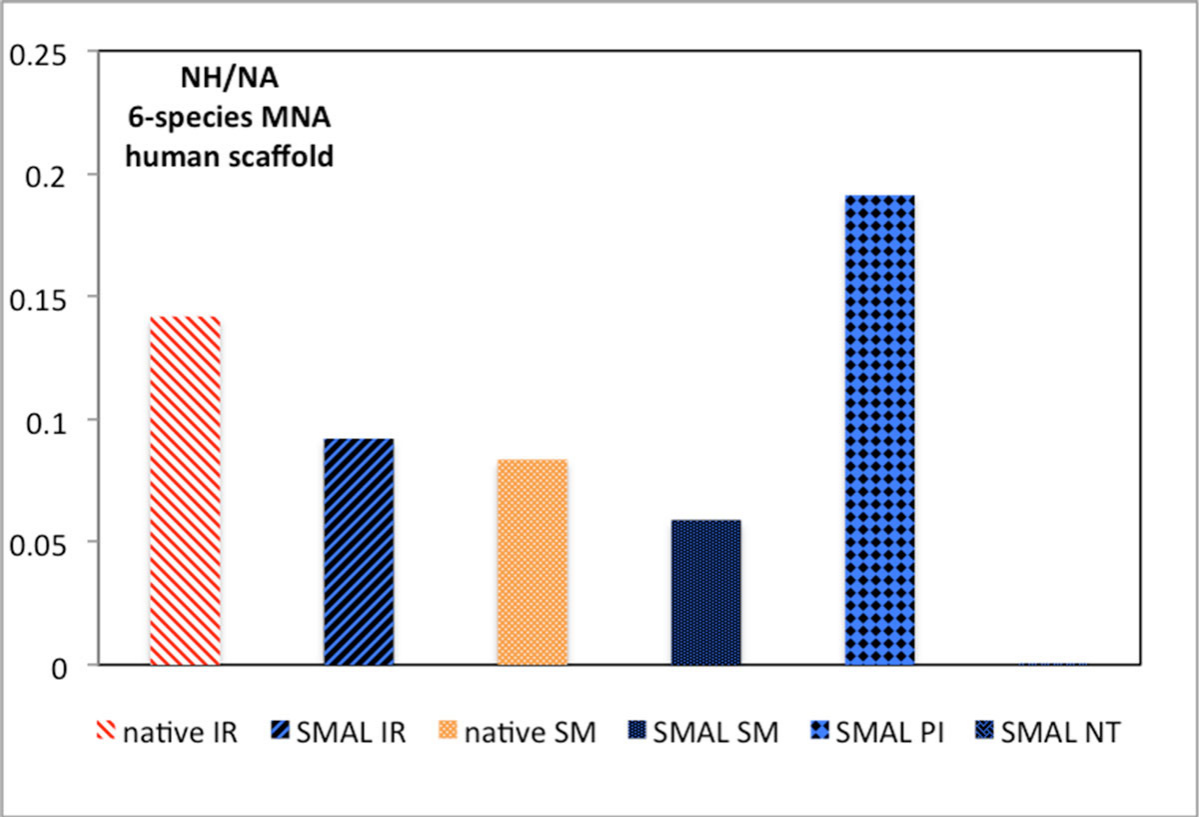
**SMAL allows creation of MNAs based on pairwise alignment algorithms that are superior to any existing native MNA algorithm for a given measure**. Alignments are shown for six species with the human PPIN as the scaffold. The same overall picture of SMAL based on PINALOG PNAs holds for 4, 5, 6, 7, 8-species MNAs as well as for any choice of the scaffold. The y-axis shows the fraction of all aligned nodes that are homologous. Higher values represent a biologically more relevant alignment. Algorithms are abbreviated: IR - IsoRankN, SM - SMETANA, PI - PINALOG, NT - NETAL. Since PINALOG and NETAL do not have native MNA implementations, there are no native data to report.

Pairwise alignment algorithms outnumber native multiple network aligners. SMAL allows any pairwise alignment algorithm to be used to produce MNAs. As outlined above, the characteristics of pairwise alignments are largely conserved in a SMAL MNA. Thus, if the characteristics of a pairwise aligner are favorable in a given research context, it becomes possible to create MNAs with similar characteristics with SMAL. PINALOG for example outperforms other network alignment algorithms considered by us in aligning homologous proteins. The average of the *NH*/*NA *measure over pairwise alignments of all eight species considered in this study was <0.01% for NETAL, 7.2% for IsoRankN, 8.1% for SMETANA and 19.6% for PINALOG respectively. A SMAL MNA based on PINALOG outperforms existing native MNAs on the same measure with *NH*/*NA=*19.1% versus 14.2% for native IsoRankN, followed by 9.2% for SMAL based on SMETANA, 8.4% for native SMETANA, 5.9% on SMAL based on SMETANA and finally <.1% for SMAL based on NETAL (Figure 5).

#### NETAL: MNAs with high numbers of conserved interactions

**Figure 6:**
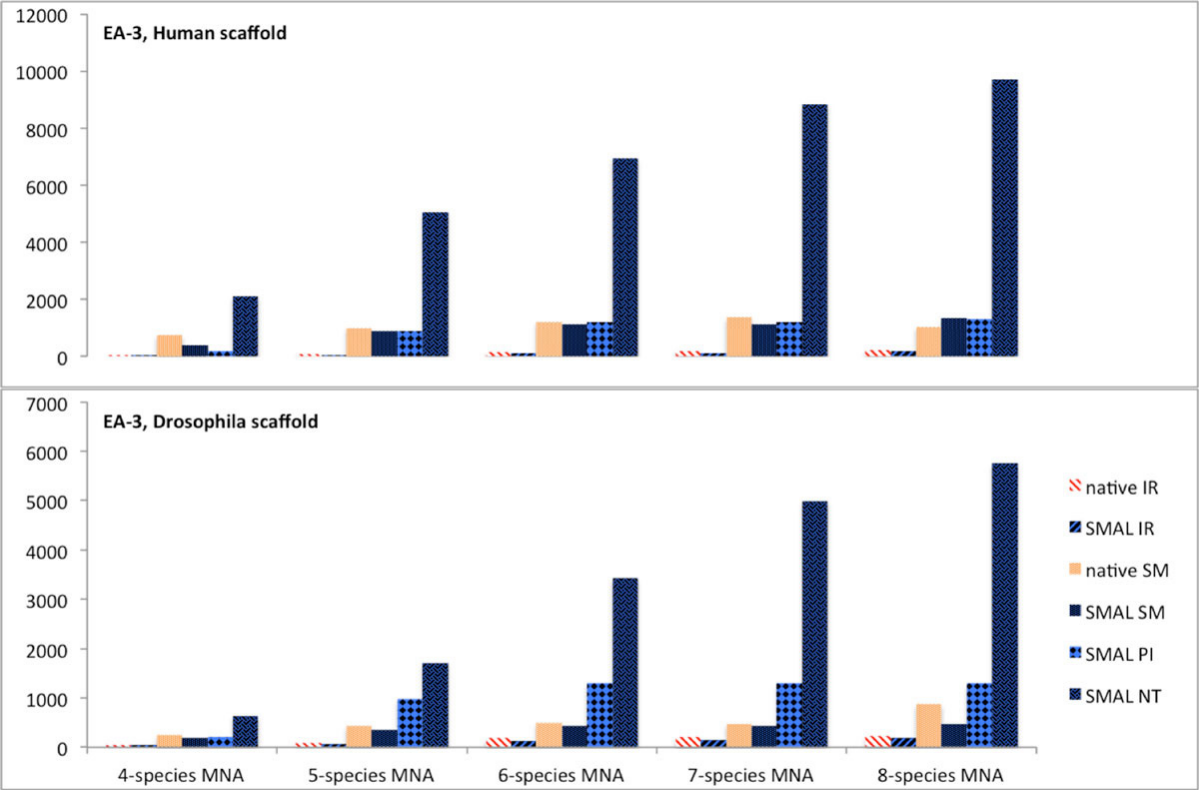
**SMAL based on NETAL**. The y-axis shows the number of interactions conserved in at least 3 species in a MNA. Each bar represents the value of this measure achieved by a MNA computed with a specific algorithm abbreviated as: IR - IsoRankN, SM - SMETANA, PI - PINALOG, NT - NETAL. SMAL MNAs based on NETAL achieve by far the highest scores. (Top) is a MNA with Human as the scaffold. (Bottom) is the same measure for MNAs using Drosophila as scaffold. While overall less interactions are conserved in at least 3 species when using Drosophila, SMAL based on NETAL again outperforms all other algorithms with SMAL based on PINALOG consistently ranking second. The native MNA implementations (available only for SMETANA and IsoRankN) as well as their SMAL counterparts achieve much lower scores. These results show that SMAL allows for creation of MNAs based on pairwise algorithms that outperform existing native MNA algorithms for specific applications or measures.

NETAL is the only algorithm in this study that does not use biological information for its alignments (e.g. BLAST bit scores for pairs of proteins) and consequently, NETAL alignments score lower on the biologically inspired measures. Yet NETAL is by far the fastest algorithm and identifies the highest number of conserved interactions in the pairwise alignments considered by us. Using NETAL with SMAL creates MNAs that maintain these valuable characteristics as shown in Figure 6.

### Speed of alignments

In this study we worked with two native multiple network aligners (SMETANA and IsoRankN) and two pairwise aligners (PINALOG and NETAL) to illustrate the efficiency aspect of several very dissimilar approaches to network alignment. Table 6 gives an overview over the key parameters and characteristics that are relevant to this study.

**Table 6:** Algorithms used in this study and their key characteristics

Algorithm	Commandline arguments	Node alignment	Notes
**IsoRankN**	--K 30 --thresh 1e-4 --alpha 0.9 --maxveclen 1000000	many-to-many	native MNA

**NETAL**	-a 0.0001 -b 0 -c 0.5 -i 2	one-to-one	no BLAST

**PINALOG**	none	primarily one-to-one	

**SMETANA**	none	many-to-many	native MNA

PINALOG and SMETANA do not require specific arguments when running the respective commandline implementations. Arguments for IsoRankN and NETAL have been used following recommendations by the authors in the literature and can be used to specify parameters that control the internal cost functions and put limits on iterations and other data that influence running time and memory requirements.

**Figure 7:**
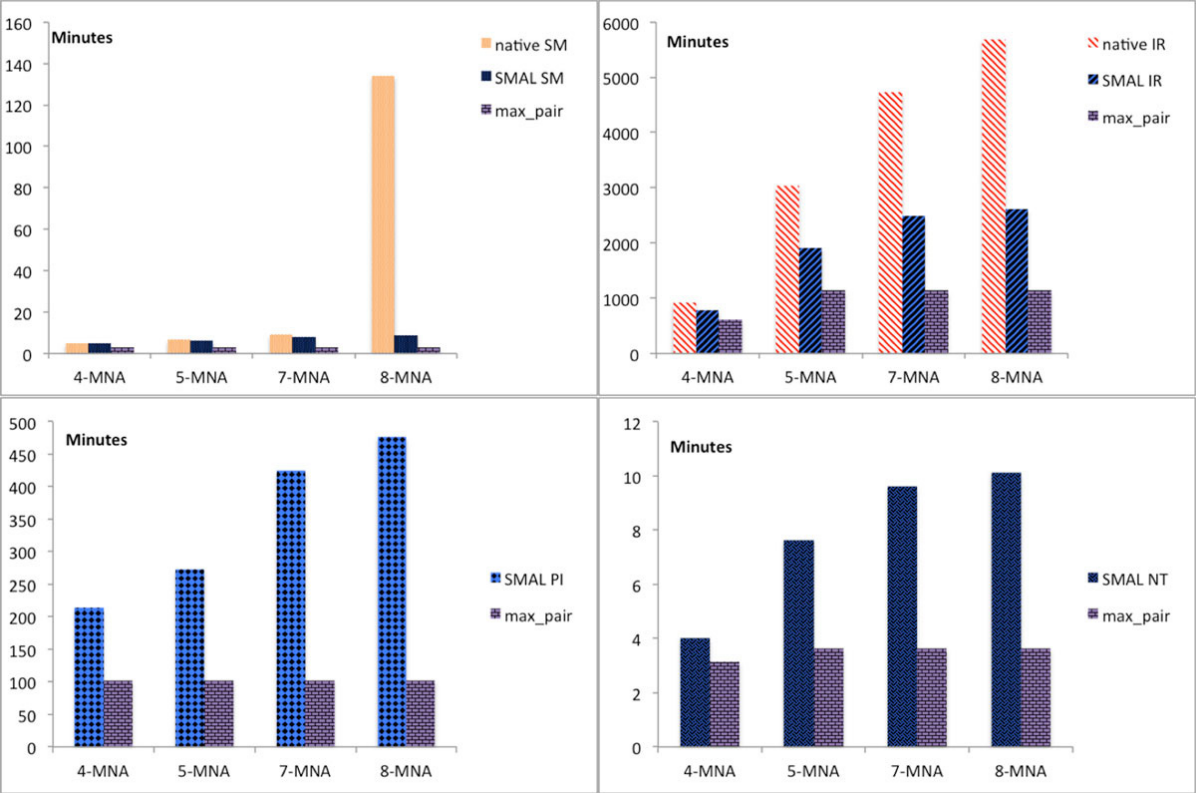
**Time-analysis of SMAL MNA creation**. The y-axis shows time to create a MNA in minutes. The four graphs represent values for different algorithms. (Top) compares native MNA creation to SMAL. (Bottom) graphs represent algorithms that do not possess native MNA versions. The small (purple brick) bars represent the most expensive pairwise alignment and show a lower bound for SMAL when pairwise alignments are parallelized. As the number of networks increases, the low complexity of SMAL emphasizes the time savings compared to native MNA algorithms. Note the differences in scale on the four graphs where total times range from less than ten minutes to several days.

Since the pairwise alignments are independent from each other, their computation can be parallelized and distributed across multiple cores or machines. Even without parallelization, SMAL outperformed native MNA alignment algorithms by large margins in our experiments, as shown in Figure 7. We note that the most computationally expensive part in this process, by far, was the creation of the pairwise alignments. This step took us from a few minutes to many hours depending on the pairwise aligner employed. By contrast, combining PNAs into a SMAL MNA including computation of the conserved edges took less than 10 seconds even for the largest alignments conducted as part of this study. All the time measurements reported in this paper were from computations conducted on a machine with dual six core 32nm Xeon processors at 3.47 GHz (hyper-threaded for 24-fold parallelism) and 86 GB of registered, ECC DDR3 RAM @1066 MHz.

## Conclusions

In this paper we introduced SMAL, a method for combining pairwise network alignments into a multiple network alignment. In contrast with other established methods, SMAL alignments are persistent in that established node correspondences do not change as additional networks are added. As the MNAs are also invariant to the order in which pairwise alignment are computed, SMAL can be enriched with additional PPINs at any point in time. This property makes the alignments suitable for iterative exploration of PPI data. SMAL is also significantly faster than other MNA algorithms and can be easily parallelized, allowing for the computation of very large MNAs covering many species. Our experiments indicate that native MNA algorithms, which are significantly slower than SMAL, may produce alignments, which, on average, score better than SMAL-based alignments produced using the pairwise versions of the same algorithms. However, SMAL allows scientists to use any of the (much larger number of) specialized pairwise alignment algorithms available today to obtain MNAs. In many cases, this leads to superior MNAs as compared to those created with native MNA algorithms.

## List of abbreviations used

MNA: Multiple Network Alignment; PNA: Pairwise Network Alignment; PPI: Protein-Protein Interaction; PPIN: Protein-Protein Interaction Network; SMAL: **S**caffold-Based **M**ultiple Network **Al**igner.

## Competing interests

There are no competing interests.

## Authors' contributions

RS proposed the formulation and the properties which constitute the key characteristics of the method, provided research guidance, and (ironically) christened the method SMAL. JD developed the SMAL algorithm, implemented it, and did the theoretical analysis. JP conducted biological analysis. The manuscript was written by RS, JD, and JP.

## Supplementary Material

Additional File 1 **Overview of PPIN alignment algorithms**. Table in landscape format; HTML, viewable in any browser; filename: 1471-2105-16-S14-S11-S1.html. Abbreviations used in the table: LP - local pairwise aligner, GP - global pairwise aligner, LM - local multiple aligner, GM - global multiple aligner, FC - functional coherence, EC - edge correctness, GOC - Gene Ontology consistency, Sp - specificity, NS - number of solutions, HP - homologene pairs, NH - number of homologene pairs, CN - correct nodes, NC - number of correct solutions. Footnotes to the table: * n1 = V1, n2 = V2, m2 = E2, m2 = E2; ** n = max{|V1|,|V2|} m = max{|E1|,|E2|} Click here for file

Additional File 2 **Pseudo-code 2 - transforming a native MNA for comparison with SMAL**. The pseudo-code outlines a method to transform a MNA obtained from a native MNA algorithm into a SMAL-like MNA. Protein alignments that are not relevant to a given scaffold are stripped and alignment clusters containing multiple scaffold proteins are duplicated. This process allows for comparison between SMAL and other MNA algorithms. Click here for file

## Declarations

This research was supported by funding from National Science Foundation grant IIS-0644418 (CAREER). Publication costs were covered in part, through a grant from the Center for Computing in Life Sciences at San Francisco State University.

This article has been published as part of *BMC Bioinformatics *Volume 16 Supplement 13, 2015: Proceedings of the 12th Annual MCBIOS Conference. The full contents of the supplement are available online at http://www.biomedcentral.com/bmcbioinformatics/supplements/16/S13.
