# Supplementary material for: Global multiple protein-protein interaction network alignment by combining pairwise network alignments
Source: BMC Bioinformatics. 2015 Sep 25;16(Suppl 13):S11. doi: 10.1186/1471-2105-16-S13-S11 (PMC4597059; doi:10.1186/1471-2105-16-S13-S11)
Supplement: Additional File 1 — Overview of PPIN alignment algorithms. Table in landscape format; HTML, viewable in any browser; filename: 1471-2105-16-S14-S11-S1.html. Abbreviations used in the table: LP - local pairwise aligner, GP - global pairwise aligner, LM - local multiple aligner, GM - global multiple aligner, FC - functional coherence, EC - edge correctness, GOC - Gene Ontology consistency, Sp - specificity, NS - number of solutions, HP - homologene pairs, NH - number of homologene pairs, CN - correct nodes, NC - number of correct solutions. Footnotes to the table: * n1 = V1, n2 = V2, m2 = E2, m2 = E2; ** n = max{|V1|,|V2|} m = max{|E1|,|E2|} [file 1471-2105-16-S13-S11-S1.html]

Overview of PPIN alignment algorithms


Overview of PPIN alignment algorithms

| Algorithm Name  (Citation) | Class | Algorithm Description | Data Sets | Algorith Speed | Quality | Public Availability |
| PathBLAST (Kelley et al., 2003) | LP | BLAST search of linear subnetworks | DIP (2002) | 7 min, with V = 5593 E = 1389 | Up to 4.1% of proteins included in alignment | www.pathblast.org/ |
| NetworkBLAST (Sharan et al., 2005) | LP | BLAST search of cluster subnetworks | DIP (2004) | Not tested | 40-52% of predictions validated experimentally | www.cs.tau.ac.il/~bnet/networkblast.html |
| MaWISH (Koyut�rk et al., 2006) | LP | Evolutionary model | BIND and DIP | Not tested | Up to 1430/15884 nodes matched | Contact authors |
| Baysean method (Berg and Lassig, 2006) | LP | Statistical approach | (Su et. al.  2004) (symatlas.gnf.org) | O(n3) | A measure of network divergence | Contact authors |
| Match and Split (Narayanan and Karp, 2007) | LP | Defined critera of correctness and efficency | MIPS | O(n1n2 + (n1 + n2)m1m2)\* | Se 25% Sp 47.5% vs Se 40.9% Sp 18.5% NetworkBLAST | Contact authors |
| Phunkee (Cootes et al., 2007) | LP | BLAST search combined with subgraph context | Database of Interacting Proteins | Not tested | 557 functionaly consistent subgraphs | www.sbg.bio.ic.ac.uk/~phunkee/ |
| AlignNemo (Ciriello et al. 2012) | LP | Iterative alignment growth combining topology and homology | DIP (2011) | <4 min V = 7548 and 5053, E = 22969 and 22254 | NS 115 NC 53 vs NS 45 NC 23 NetworkBLAST | www.bioinformatics.org/alignnemo |
| IsoRank (Singh et al., 2008) | GP | Eigenvalue formulation | Combined DIP, BioGRID and HPRD | Not tested | FC score of 0.220 vs 0.223 of Homologene | groups.csail.mit.edu/cb/mna/ |
| GRAAL (Kuchaiev et al., 2010) | GP | Greedy Heuristic: node topological and sequence similarity | (Radivojac et al. 2008) and (Collins et al. 2008) | Not tested | EC score of 0.117 vs 0.0389 of IsoRank | bio-nets.doc.ic.ac.uk/GRAAL\_suppl\_inf/ |
| MI-GRAAL (Kuchaiev and Pržulj, 2011) | GP | Automatically selects from multiple node topological similarity measures | (Peregrin-Alvarez et al., 2009) and (Parrish et al., 2007) | O(|n1| × (m1 + |n1| × log(|n1|))\* | EC score of 0.23 vs .0389 of IsoRank | bio-nets.doc.ic.ac.uk/MI-GRAAL/ |
| PISwap (Chindelevitchet al., 2010) | GP | Greedy Heuristic: manual balance of node topo. and seq. similairty | (Zalavskiy et al 2009) | 10 min sum(V) = 14254 sum(E) = 42361 | FC score of 0.510 vs 0.519 of IsoRank | omictools.com/piswap-s5588.html |
| PATH/GA (Zaslavskiyet al. 2009) | GP | Relaxation of cost function | (Bandyopadhyay et al 2006) | < 30 minutes with V = 4389 and 7038, E = 14319 and 20720 | 41 HPs vs 39 HPs of IsoRank | jean-philippe.vert@mines-paristech.fr |
| SPINAL (Aladag and Erten, 2013) | GP | Pairwise neighborhood matching and iterative growth | IsoBase (Park et al., 2011) | 49 min, with V = 5499 and 9633, E = 31261 and 34327 | GOC comperable to IsoRank | code.google.com/p/spinal/ |
| NETAL (Behnam Neyshabur et al. 2013) | GP | Alignment score matrix combined with a greedy approach | Radivojac et al. (2008) to the Collins et al. (2008) | O(m2)=O(n2log2n) \*\* | EC score of 0.361 vs 0.0389 for IsoRank | bioinf.modares.ac.ir/software/netal/ |
| PINALOG (Phan and Sternberg 2012) | GP | Combines sequence, function and topology data | IntAct | < 24 hours V = 5674 and 9003, E = 49830 and 34935 | NH score of 454 vs 165 of IsoRank | www.sbg.bio.ic.ac.uk/~pinalog/ |
| NetworkBlast-M (Mu-Fen Hsieh and Sing-Hoi Sze, 2010) | LM | NetworkBLAST expanded to MNA | IntAct, DIP and SNDB | 27 hours for sum(V) = 10339 with sum(E) = 64890 | Sp 94.4%, Se  46.2 vs Sp 96.5%, Se 37.75 for MaWISH | www.cs.tau.ac.il/~bnet/License-nbm.html |
| Submap (Ayet al., 2011) | LM | Subnetwork mapping combined with MWIS algorithm | KEGG (Ogata et al., 1999) | < 20 min for pathways up to E < 140 and subnetowrk size < 4 | Up to 44.2% alternative subnetworks | bioinformatics.cise.ufl.edu/SubMAP.html |
| IsoRankN (Liao et. al. 2009) | GM | Spectral clustering | DIP, BioGRID and HPRD combination | 7 hour pre-processing time, < 5 min alignment sum(V) = 87737 sum(E) = 98945 | GO/KEGG of 623/2200 vs 478/1551 for IsoRank | groups.csail.mit.edu/cb/mna/ |
| SMETANA (Mohammad et. al. 2013) | GM | Probabilistic scoring combined with a greedy approach | NAPAbench and IsoBase (DIP, BioGRID and HPRD) | < 2 min for up to 8 networks of 1000 nodes | Sp 87.04% CN 6349 vs Sp 64.5% CN 4069 for IsoRank | www.ece.tamu.edu/~bjyoon/SMETANA/ |
| Graemlin 2.0 (Flannick et al., 2009) | GM | Trainable hill-climb algorithm | DIP, SNDB and KEGG | < 1 min or O(b x (n + m)) where b (number of iterations) < 10 | Sp 81% CN 350 vs Sp 62% CN 350 IsoRank | graemlin.stanford.edu/download.php |
|  | | | | | | |
| Abbreviations: |  | EC - edge correctness | Sp - specificity | HP - HomoloGene Pairs | CN - Correct Nodes | \* n1 = V1 , n2 = V2 , m2 = E2 , m2 = E2 |
| FC - functional coherence |  | GOC - Gene Ontology Consistency | NS - number of solutions | NH - Number of Homologene pairs | NC - number of correct solutions | \*\* n = max{|V1|,|V2|} m = max{|E1|,|E2|} |
