# Supplementary material for: Global multiple protein-protein interaction network alignment by combining pairwise network alignments
Source: BMC Bioinformatics. 2015 Sep 25;16(Suppl 13):S11. doi: 10.1186/1471-2105-16-S13-S11 (PMC4597059; doi:10.1186/1471-2105-16-S13-S11)
Supplement: Additional File 2 — Pseudo-code 2 - transforming a native MNA for comparison with SMAL. The pseudo-code outlines a method to transform a MNA obtained from a native MNA algorithm into a SMAL-like MNA. Protein alignments that are not relevant to a given scaffold are stripped and alignment clusters containing multiple scaffold proteins are duplicated. This process allows for comparison between SMAL and other MNA algorithms. [file 1471-2105-16-S13-S11-S2.pdf]

Pseudo-code 2: native MNA related to scaffold PPIN

Designate scaffold PPIN G_s
Create native MNA
$A' \leftarrow multiple\_network\_alignment(G_1, G_2, \ldots, G_n)$
Extract alignment clusters containing nodes of the scaffold PPIN
Initialize $V^{native} = \emptyset$
For each node of G_s, $v \in V_s$:
    Initialize $\Gamma(v) = \{v\}$
    For each node alignment cluster of the native MNA $C(v')$ for all $v' \in V'$:
        if $v \in C(v')$:
            $\Gamma(v) \leftarrow \Gamma(v) \cup C(v')$
    $V^{native} \leftarrow V^{native} \bullet \Gamma(v)$ # concatenate sets
Conserved edges equivalent to pseudo-code 1
